# Protective Effects of *Euphrasia officinalis* Extract against Ultraviolet B-Induced Photoaging in Normal Human Dermal Fibroblasts

**DOI:** 10.3390/ijms19113327

**Published:** 2018-10-25

**Authors:** Ying Liu, Eunson Hwang, Hien T. T. Ngo, Haribalan Perumalsamy, Yeon Ju Kim, Lu Li, Tae-Hoo Yi

**Affiliations:** College of Life Sciences, Kyung Hee University, 1732, Deogyeong daero, Giheung-gu, Yongin-si, Gyeonggi-do 17104, Korea; y.liu0915@khu.ac.kr (Y.L.); firsthes@khu.ac.kr (E.H.); hienngo211@khu.ac.kr (H.T.T.N.); harijai2004@gmail.com (H.P.); yeonjukim@khu.ac.kr (Y.J.K.); lilu@khu.ac.kr (L.L.)

**Keywords:** *Euphrasia officinalis*, ultraviolet B, photoaging, matrix metalloproteinases, type I procollagen, apoptosis

## Abstract

Ultraviolet (UV) radiation induces skin photoaging, which is associated with the elevation of matrix metalloproteinases (MMPs) and the impairment of collagen. The Euphrasia species play a well-known role in the treatment of certain eye disorders through their anti-oxidative and anti-inflammatory activities. However, their protective activity toward UVB-induced damage remains unclear. In the present study, we investigated the protective effect of *Euphrasia officinalis* (95% ethanol extract) on UVB-irradiated photoaging in normal human dermal fibroblasts (NHDFs). Our results show that *Euphrasia officinalis* extract exhibited obvious reactive oxygen species (ROS) and 2,2′-azino-bis (3-ethylbenzothiazoline-6-sulfonic acid) (ABTS) radical scavenging activity, enhanced NHDF cell migration, and reduced UVB-induced apoptosis. The UVB-induced increases in MMP-1 and MMP-3 and decrease in type I procollagen were ameliorated by *Euphrasia officinalis* treatment, which worked by suppressing the mitogen-activated protein kinase (MAPK) and nuclear transcription factor activator protein 1 (AP-1) signaling pathways. Taken together, our data strongly suggest that *Euphrasia officinalis* ethanol extract could reduce UVB-induced photoaging by alleviating oxidative stress, proinflammatory activity, and cell apoptosis.

## 1. Introduction

Skin aging is a complex process that is affected by both intrinsic factors such as genetics, hormonal changes, and metabolic processes and extrinsic factors such as solar radiation, smoking, pollution, and chemical exposure [1,2]. Among the various factors, solar ultraviolet (UV) radiation is a major extrinsic photoaging factor that is divided into three categories: UVA (315–400 nm), UVB (280–315 nm), and UVC (100–280 nm). UVA and UVB can penetrate the Earth’s atmosphere and cause sunburn, photoaging, and some forms of skin cancer in humans [3]. Cumulative exposure to UVB evokes photoaging, which is manifested by the formation of deep wrinkles, pigmentation, and dry skin [4].

UVB is mostly absorbed by the epidermis, but it can also reach the upper part of the dermis [5,6]. Severe and chronic UVB irradiation causes photoaging by the production of excessive intracellular reactive oxygen species (ROS); this results in oxidative stress, which is an important mediator of damage to cell structures [7]. UVB-induced ROS generation activates the mitogen-activated protein kinase (MAPK) signaling pathway [8]. The MAPKs are a family of serine/threonine-specific protein kinases: the c-Jun N-terminal kinase (JNK), extracellular signal-regulated kinase (ERK), and p38 kinase [9]. MAPK activation upregulates the transcription of activator protein 1 (AP-1), which induces matrix metalloproteinase (MMP) expression and collagen degradation. Specifically, MMP-1, an interstitial collagenase or fibroblast collagenase, degrades transforming growth factor-β1 (TGF-β1), collagen, and elastin in the extracellular matrix (ECM) [10]. Furthermore, inflammation and apoptosis have been proposed as the major hallmarks of skin photoaging [11,12]. The activation of MAPKs upregulates nuclear factor-κB (NF-κB), which induces the secretion of the proinflammatory cytokine interleukin-6 (IL-6). An increase in IL-6 leads to collagen degradation by accelerating fibroblast and MMP expression and cell apoptosis.

*Euphrasia officinalis*, commonly known as eyebright, is an annual, herbaceous, semi-parasitic plant that is part of the family Orobanchaceae (formerly assigned to Scrophulariaceae). The plant is found throughout Europe, Asia, and North America. It has traditionally been used as an eye lotion in the treatment and prevention of eye disorders such as conjunctivitis, ophthalmia, styes, and ocular allergies [13,14]. From a phytochemical point of view, *E. officinalis* contains many bioactive compounds, such as iridoids, flavonoids, phenolic acids, and etheric oils [15,16]. Prior pharmacological investigations of *E. officinalis* extracts have revealed its anti-hyperglycemic [17], antioxidant [18], anti-inflammatory [19], and antimicrobial [20] properties. A recent study found that an eye drop containing *Matricaria chamomilla* and *E. officinalis* extracts conferred protective properties against UVB-induced inflammation and oxidative stress in human corneal epithelial cells [21]. Although *E. officinalis* is widely known for its many pharmacological actions, its effects on skin aging have not been investigated. Therefore, we investigated the efficacy of an ethanol extract of *E. officinalis* on UVB-irradiation-induced photoaging in normal human dermal fibroblasts (NHDFs).

## 2. Results

### 2.1. Identification of Components by Ultra-High Performance Liquid Chromatography-Quadrupole Time-of-Flight Mass Spectrometry (UPLC-QTOF-MS) Analysis

In previous literatures, the high performance liquid chromatography (HPLC) and liquid chromatography–mass spectrometry (LC-MS)/MS methods were used to identify phenolics, flavonoids, and acteoside in *E. officinalis* [18,22]. In our ultra-high performance liquid chromatography-quadrupole time-of-flight mass spectrometry (UPLC-QTOF-MS) analysis, we tentatively identified caffeic acid, luteolin-glucoside, rutin, and acteoside, as shown in Figure S1 and Table S1.

### 2.2. Effect of E. officinalis on Cell Viability

**Figure 1:**
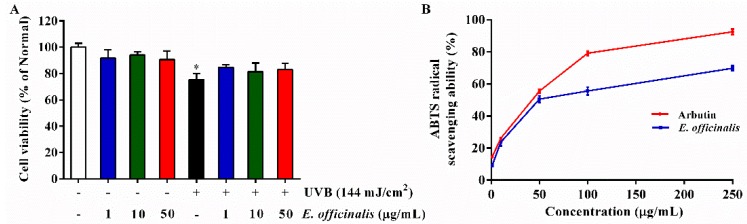
Effect of *E. officinalis* on cell viability and ABTS (2,2′-azino-bis (3-ethylbenzothiazoline-6-sulfonic acid)) radical scavenging ability. (**A**) Cell viability after 72 h with or without ultraviolet B (UVB) (144 mJ/cm^2^) and *E. officinalis* (1 µg/mL, 10 µg/mL, or 50 μg/mL); (**B**) ABTS radical scavenging ability of *E. officinalis*. Arbutin was used as a positive control. Values are means ± standard deviation (SD). * and # indicate significant differences between the non-irradiated control and the UVB-irradiated control, respectively. * *p* < 0.05 versus the non-irradiated control.

NHDFs were exposed to UVB irradiation (144 mJ/cm^2^) and then treated with *E. officinalis* (1 µg/mL, 10 µg/mL, or 50 µg/mL). Following UVB exposure, NHDF density decreased noticeably, with a cell viability only 75.3% (Figure 1A) of the normal control. At the indicated concentrations, *E. officinalis* had no significant cytotoxic effect on the cells. In fact, treatment with *E. officinalis* improved the cell viability of UVB-irradiated NHDFs.

### 2.3. 2,2′-Azino-bis (3-ethylbenzothiazoline-6-sulfonic acid) (ABTS) Radical Scavenging Activity of E. officinalis

An ABTS (2,2′-azino-bis (3-ethylbenzothiazoline-6-sulfonic acid)) radical scavenging capacity assay was carried out to evaluate the antioxidant properites of E. officinalis. As shown in Figure 1B, *E. officinalis* produced a dose-dependent increase in ABTS radical-scavenging activity, with an IC_50_ value of 49.8 µg/mL. At 250 µg/mL, E. officinalis inhibited ABTS radicals by 72.8%.

### 2.4. Effects of E. officinalis on Intracellular Reactive Oxygen Species (ROS) Production

**Figure 2:**
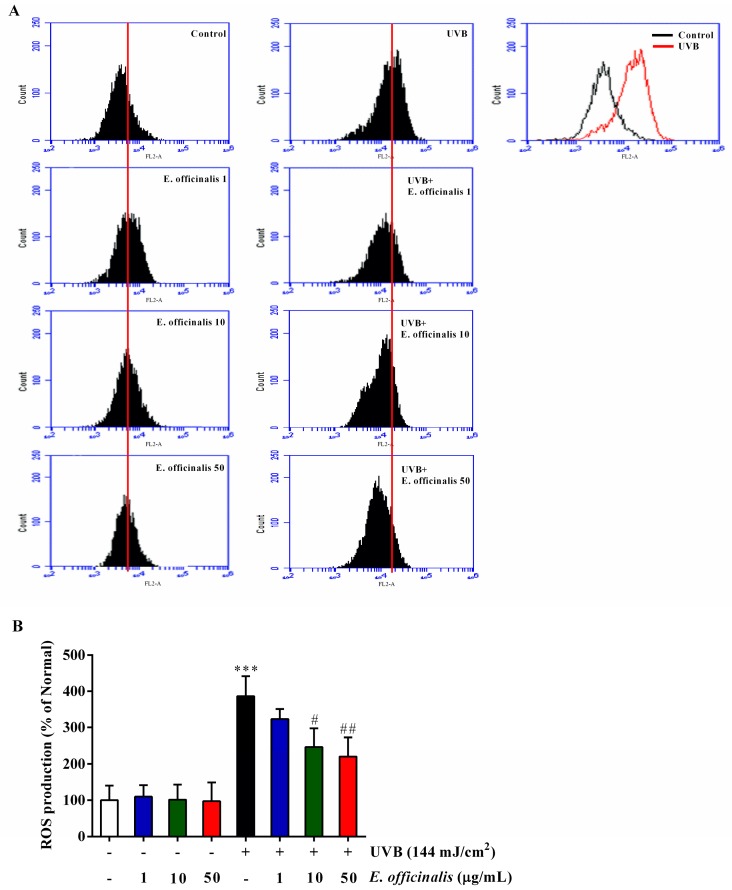
Effect of *E. officinalis* on the generation of reactive oxygen species (ROS) in UVB-irradiated normal human dermal fibroblasts (NHDFs). (**A**) ROS levels in NHDFs were determined after 24 h of UVB radiation with and without *E. officinalis* treatment. The number of cells is plotted versus the dichlorofluorescein fluorescence detected by the FL-2 channel; (**B**) The relative ROS generated by NHDFs is shown. Values are means ± SD. *** *p* < 0.001 versus the non-irradiated control. # *p* < 0.05 and ## *p* < 0.01 versus the UVB-irradiated control.

As shown in the flow cytometric analysis in Figure 2, UVB irradiation increased the production of intracellular ROS compared to untreated control cells. Management with *E. officinalis* at doses of 10 µg/mL and 50 µg/mL decreased intracellular ROS production by 36.3% and 43.0%, respectively, compared to untreated UVB-irradiated NHDF cells.

### 2.5. Effect of E. officinalis on Normal Human Dermal Fibroblast (NHDF) Cell Migration

**Figure 3:**
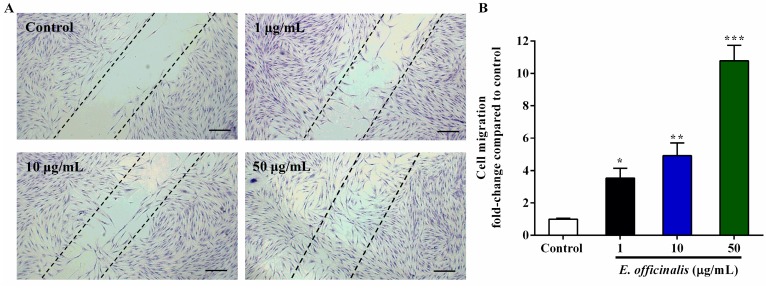
Effect of *E. officinalis* on NHDF cell migration as determined by a wound-healing assay. (**A**) NHDF cells cultured for 72 h were fixed and stained with crystal violet. The dotted lines represent the wound boundary of Control. Magnification scale: 100×. Scale bar: 200 µm; (**B**) Graphical presentation of wound-healing assay. Values are means ± SD. * *p* < 0.05, ** *p* < 0.01, and *** *p* < 0.001 versus the untreated control.

The effect of *E. officinalis* on NHDF cell migration was detected using a wound-healing assay. As seen in Figure 3, *E. officinalis* dose-dependently accelerated the migration of NHDF cells. In untreated control cells, NHDF migration was barely detectable after 72 h of incubation, whereas *E. officinalis* greatly enhanced the migratory activity and left significant migration tracks, especially at 50 µg/mL.

### 2.6. Effect of E. officinalis on NHDF Cell Apoptosis

**Figure 4:**
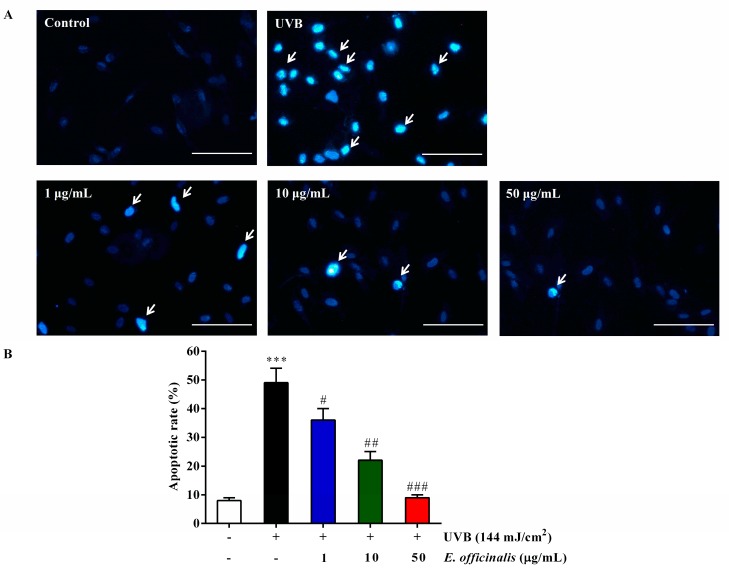
Anti-apoptotic effect of *E. officinalis* on UVB-irradiated NHDFs. (**A**) Apoptotic cells were detected by Hoechst 33258 staining. Cells that underwent apoptosis are indicated by arrows. Magnification scale: 400×. Scale bar: 10 µm; (**B**) Apoptotic rate was calculated as a percentage of apoptotic cells over the total number of cells numbers in five randomly selected areas. Values are means ± SD. *** *p* < 0.001 versus the non-irradiated control. # *p* < 0.05, and ## *p* < 0.01, and ### *p* < 0.001 versus the UVB-irradiated control.

Hoechst 33258 staining was used to observe the effect of *E. officinalis* on NHDF cell apoptosis 72 h after exposure to 144 mJ/cm^2^ of UVB radiation. The results showed that UVB significantly induced typically apoptotic nuclear morphology including nuclei fragmentation, shrinkage, and intense fluorescence owing to damage to the outer membrane (Figure 4). However, cell apoptosis was prevented after treatment with *E. officinalis*. This result clearly suggested *E. officinalis* inhibited apoptosis of NHDF cells induced by UVB-irradiation.

### 2.7. Effect of E. officinalis on MMP-1, MMP-3, IL-6, TGF-β1, and Type I Procollagen Production

**Figure 5:**
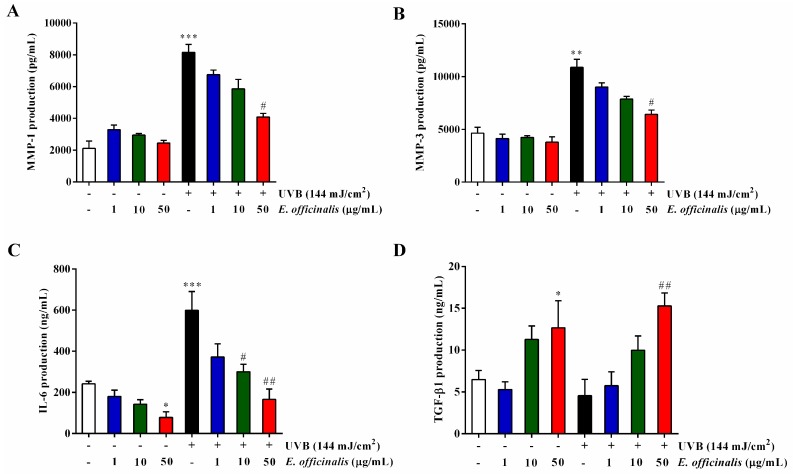

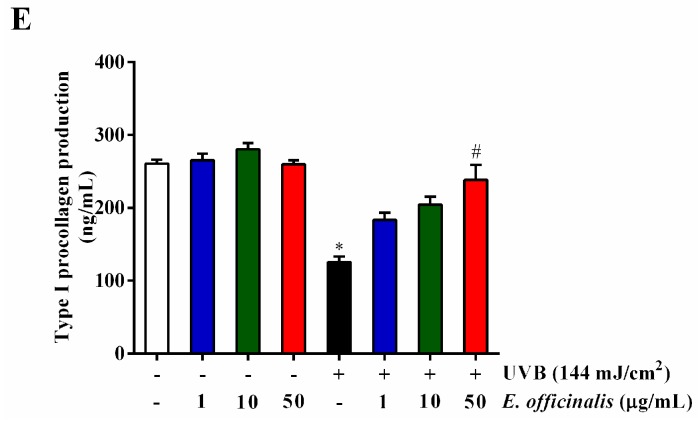
Effect of *E. officinalis* on the secretion of the matrix metalloproteinases (MMPs) and type I procollagen. The production of (**A**) MMP-1, (**B**) MMP-3, (**C**) interleukin-6 (IL-6), (**D**) transforming growth factor-β1 (TGF-β1), and (**E**) type I procollagen in non- and UVB-irradiated NHDFs. Values are means ± SD. * *p* < 0.05, ** *p* < 0.01, and *** *p* < 0.001 versus the non-irradiated control. # *p* < 0.05 and ## *p* < 0.01 versus the UVB-irradiated control.

The MMPs are a family of calcium-dependent, zinc-containing endopeptidases that can degrade all kinds of proteins in the ECM. After UVB irradiation, the secretion of MMP-1 and MMP-3 increased to 287.1% and 134.5% of that found in normal NHDF cells, respectively (Figure 5A,B). Treatment with 50 μg/mL of *E. officinalis* extract after exposure to UVB suppressed the expression of MMP-1 and MMP-3 by 49.9% and 41.1%, respectively. UVB irradiation also induced a 148.0% increase in the pro-inflammatory cytokine IL-6. Interestingly, *E. officinalis* treatment significantly inhibited that elevation, by 72.3% at 50 μg/mL (Figure 5C).

We also found that *E. officinalis* recovered the collagen degradation and TGF-β1 decrease induced in NHDFs by UVB-irradiation. In the UVB-irradiated cells, type I procollagen secretion decreased by 51.8% compared to untreated control cells (Figure 5E). However, *E. officinalis* (50 μg/mL) significantly reversed the decrease by 90.0%. Besides, TGF-β1 production reduced 29.8% by UVB-irradiation although it was hard to reach significant changes (Figure 5D). But what was interesting, *E. officinalis* (50 μg/mL) greatly improved TGF-β1 secretion by 235.8%, compared to UVB-irradiated control.

### 2.8. Effect of E. officinalis on the Expression of MMP-1 and Type I Procollagen

**Figure 6:**
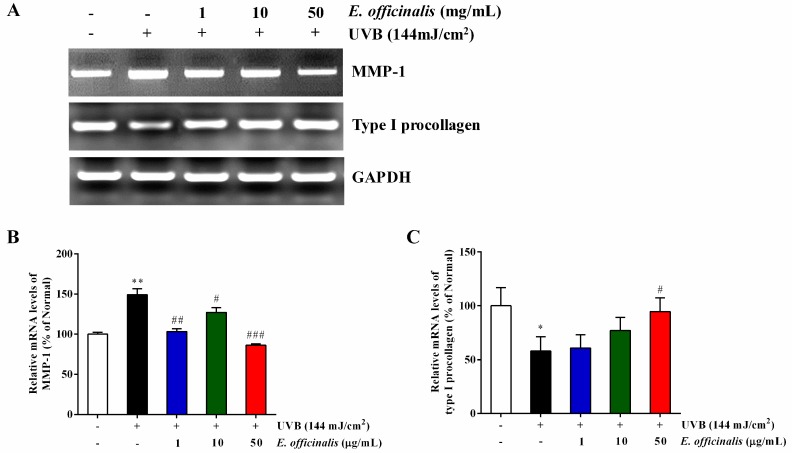
Effect of *E. officinalis* on skin aging–related mRNA expression. (**A**) mRNA expression of MMP-1 and type I procollagen in UVB-irradiated NHDFs. The mRNA levels of (**B**) MMP-1 and (**C**) type I procollagen were quantified and normalized to the corresponding glyceraldehyde-3-phosphate dehydrogenase (GAPDH) value. Densitometry data are expressed as the percentage relative to the level in the non-irradiated control and shown as the mean ± SD. * *p* < 0.05 and ** *p* < 0.01 versus the non-irradiated control. # *p* < 0.05, ## *p* < 0.01, and ### *p* < 0.001 versus the UVB-irradiated control.

We used RT-PCR analyses to determine the influence of *E. officinalis* on the mRNA expression of MMP-1 and type I procollagen in UVB-exposed NHDFs (Figure 6). Consistent with the ELISA results (Figure 5A,E), UVB exposure enhanced MMP-1 mRNA expression by 49.3% and weakened type I procollagen mRNA levels by 41.9%. After a high dose of *E. officinalis* (50 μg/mL), the mRNA expression of MMP-1 decreased by 42.2%, and that of type I procollagen increased by 63.2%.

### 2.9. Effect of E. officinalis on Activator Protein 1 (AP-1) Transcription

**Figure 7:**
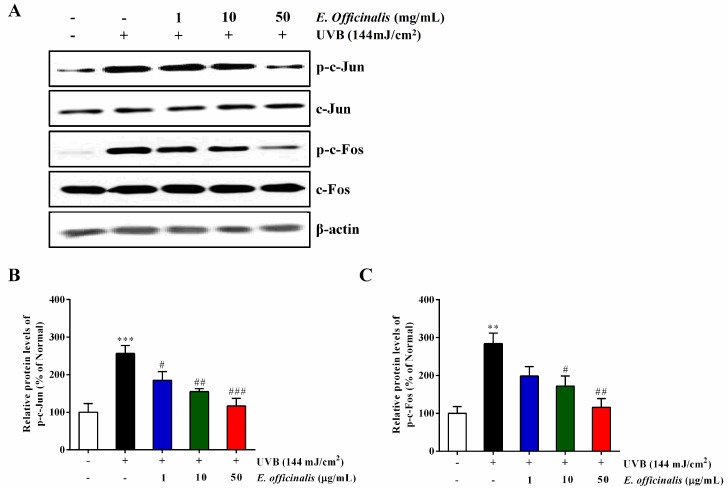
Effect of *E. officinalis* on activator protein 1 (AP-1) signaling proteins. (**A**) The protein levels of p-c-Jun and p-c-Fos in UVB-irradiated NHDFs were measured by Western blot analysis. The signal intensities for (**B**) p-c-Jun and (**C**) p-c-Fos were quantified and normalized to the corresponding β-actin value. Densitometry data are expressed as the percentage relative to the non-irradiated control and shown as the mean ± SD. ** *p* < 0.01 and *** *p* < 0.001 versus the non-irradiated control. # *p* < 0.05, ## *p* < 0.01, and ### *p* < 0.001 versus the UVB-irradiated control.

AP-1 is a vital downstream effector of the MAPK signaling pathway. Its family members include the c-Jun and c-Fos proteins. AP-1 regulates the production of MMPs, which control degradation in the ECM [23]. We used the Western blot technique to explore the inhibitive activity of *E. officinalis* on MMP levels through AP-1 transcription levels. As shown in Figure 7, UVB exposure enhanced the phosphorylation of both c-Jun (p-c-Jun) and c-Fos (p-c-Fos) by 275.4% and 397.8%, respectively. However, 50 μg/mL of *E. officinalis* treatment suppressed p-c-Jun by 52.0% and p-c-Fos by 60.8% compared to UVB-treated cells.

### 2.10. Effect of E. officinalis on the MAPK Signaling Pathway

**Figure 8:**
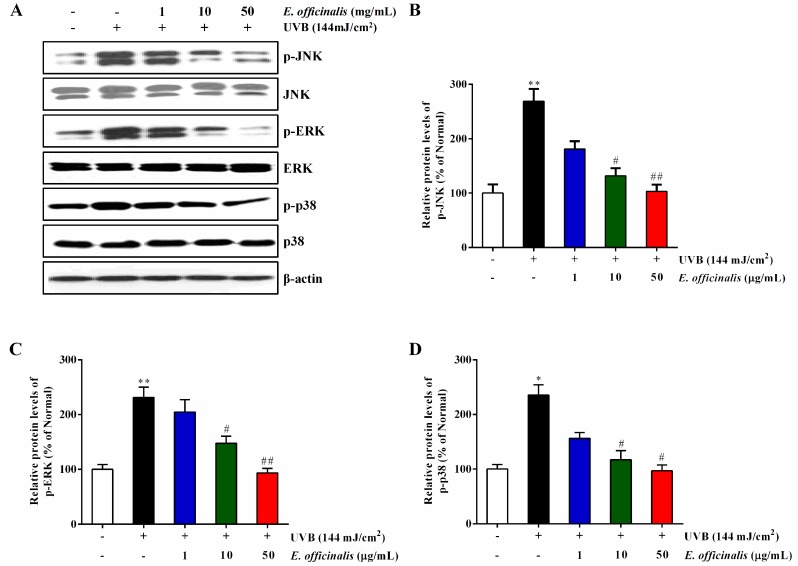
Effect of *E. officinalis* on mitogen-activated protein kinase (MAPK) signaling–related proteins. (**A**) The protein levels of the phosphorylation of c-Jun N-terminal kinase (p-JNK), extracellular signal-regulated kinase (p-ERK), and p38 (p-p38) in UVB-irradiated NHDFs were measured by Western blot analysis. The band intensities for (**B**) p-JNK, (**C**) p-ERK, and (**D**) p-p38 were quantified and normalized to the corresponding β-actin value. Densitometry data are expressed as the percentage relative to the non-irradiated control and shown as the mean ± SD. * *p* < 0.05 and ** *p* < 0.01 versus the non-irradiated control. # *p* < 0.05 and ## *p* < 0.01 versus the UVB-irradiated control.

The activation of the MAPK signal transduction pathway affects AP-1 activation in a way that upregulates the production of the MMPs [24]. MMP-1 expression leads to collagen decomposition in the connective tissues, which ultimately causes reduced elasticity and wrinkle formation [25]. To understand the inhibitory actions of *E. officinalis* extract on MMP-1 expression, we investigated whether *E. officinalis* inhibits the phosphorylation of the upstream MAPKs. The levels of phosphorylated JNK (p-JNK), ERK (p-ERK), and p38 (p-p38) proteins were significantly elevated in the UVB-irradiated group compared to normal cells, and those elevations were attenuated by *E. officinalis* (Figure 8). The original Western blot images related to this article in triplicate have been shown in Figure S2.

## 3. Discussion

The genus Euphrasia includes approximately 450 species. It is commonly known as eyebright and used to treat certain eye conditions [26]. Eyebright has an anti-inflammatory property that is also applied to relieve colds, coughs, sinus infections, sore throats, and hay fever [27,28]. The active principles include iridoids, flavonoids, and phenolic acids and display antioxidant, anti-inflammatory, anticancer, antihepatotoxic, and antimicrobial properties [26]. These components make eyebright a potential therapeutic agent for a wide range of diseases. In this study, we identified phenolics (caffeic acid) and flavonoids (luteolin-glucoside, rutin) in a UPLC-QTOF-MS analysis of an ethanol extract of *E. officinalis* leaves.

Excessive exposure to UV rays, such as UVB radiation, is a main risk factor for skin health. One of the primary causes of photoaging induced by UVB irradiation is ROS generation. Although the skin manages ROS with its own self-defense system, excessive and chronic exposure to UVB can break down that system, leading to oxidative damage [29]. It is reported that UVB radiation elicits a strong apoptosis reaction in many cell types, including both tumor and normal cells [30]. According to a previous study, UVB-induced oxidative stress and inflammation concomitantly cause cell apoptosis [31]. In this study, 144 mJ/cm^2^ of UVB resulted in significantly decreased cell viability and remarkable cell apoptosis. Treatment with *E. officinalis* did not cause obvious alterations to cell viability, but it did significantly prevent cell apoptosis following UVB irradiation. It is worth remembering that damaged cells that evade the apoptotic process can cause tumorigenesis [11]. Therefore, the mechanism and role of the protective apoptotic signaling pathway will be the subject of a subsequent investigation. Furthermore, NHDFs play a key role in healing skin wounds. They proliferate and migrate to the wound surface where they synthesize ECM [32]. We found that *E. officinalis* markedly improved the migratory ability of NHDFs.

UVB-generated ROS and free radicals produce wrinkles and reduce the synthesis of collagen and elastic fibers [4]. Drugs derived from natural products can eliminate ROS and free radicals to prevent UVB-induced photoaging with few or no side effects [33]. A previous study suggested that a methanol extract of *Euphrasia officinalis* L. exhibited excellent antioxidant activity that correlated with its high phenolic content [18]. In this study, we have shown that an ethanol extract of *E. officinalis* exhibits antioxidant properties by scavenging ABTS radicals.

The MMPs are a family of Ca^2+-^dependent, Zn^2+^-containing endopeptidases that can break down most proteins within the ECM [34]. UVB irradiation and ROS production can activate MMP-1, which then cleaves all types of collagen and reduces the secretion of TGF-β1 [35]. TGF-β1 is a key cytokine that is involved in the synthesis of type I procollagen [36]. In our study, UVB-irradiation decreased TGF-β1 secretion, although without significant change. However, what was interesting was that *E. officinalis* (50 μg/mL) greatly promoted TGF-β1 secretion in UVB-irradiated cells. Furthermore, UVB irradiation increased the production of MMP-1 and MMP-3, whereas decreased the production of type I procollagen. However, we found that *E. officinalis* (50 μg/mL) downregulated MMP-1 and MMP-3 secretion and upregulated type I procollagen synthesis in UVB-exposed NHDFs. The effects of *E. officinalis* on the mRNA expression levels of type I procollagen and MMP-1 were also confirmed by RT-PCR analyses.

UVB irradiation induces oxidative damage by triggering the MAPK signaling pathway through the phosphorylation of the JNK, ERK, and p38 signaling cascades [37]. The activation of the MAPKs activates the transcription of AP-1 and NF-κB [31]. The increased phosphorylation of c-Jun and c-Fos increases the secretion of MMPs, which weakens the production of type I procollagen [38]. Furthermore, the transcription of NF-κB stimulates the accumulation of pro-inflammatory cytokine IL-6, causing inflammation in the skin [31]. We have here found that *E. officinalis* effectively healed UVB-induced damage by suppressing the MAPK and AP-1 signaling transduction pathways, which were confirmed by showing the inhibition of MMP-1, MMP-3, and IL-6 production and the promotion of type I procollagen and TGF-β1 synthesis.

## 4. Materials and Methods

### 4.1. Chemicals

Dulbecco’s modified Eagle’s medium (DMEM), penicillin-streptomycin, and fetal bovine serum (FBS) were all purchased from Gibco (Grand Island, NY, USA). 2,2′-azino-bis (3-ethylbenzothiazoline-6-sulfonic acid) diammonium salt (ABTS), potassium persulfate, 3-(4,5-dimethylthiazol-2-yl)-2,5-diphenyltetrazolium bromide (MTT), and dimethyl sulfoxide (DMSO) were purchased from Sigma-Aldrich (St. Louis, MO, USA). An enzyme-linked immunosorbent assay (ELISA) kit for type I procollagen was purchased from Takara (Takara, Shiga, Japan). The ELISA kits for MMP-1, MMP-3, IL-6, and TGF-β1 were purchased from R&D Systems (Minneapolis, MN, USA). Antibodies against p-c-Fos (sc-81485), c-Fos (sc-52), p-c-Jun (sc-822), c-Jun (sc-1694), and β-actin (sc-47778) and secondary antibodies conjugated to horseradish peroxidase were obtained from Santa Cruz Biotechnology (Santa Cruz, CA, USA), and p-ERK (9101s), ERK (9102s), p-JNK (9251s), and JNK (9252s) were obtained from Cell Signaling Technology (Danvers, MA, USA).

### 4.2. Sample Preparation

We used 95% ethanol extracts of *Euphrasia officinalis* (dried leaves) obtained from Mountain Rose Herbs (Eugene, OR, USA). The dried and pulverized *E. officinalis* was weighed (10 g) and then extracted three times with 50 mL of 95% ethanol for 24 h at 37 °C. All of the extracts were combined and filtered, and then the solvent was completely evaporated using a rotary vacuum evaporator at 40 °C. The total yield was 1.1 g of crude extract.

### 4.3. UPLC-QTOF-MS Analysis

For the UPLC-QTOF-MS analysis, the dried crude extract was dissolved with methanol (5 mg/mL) and filtered with a 0.22-μm membrane filter. The chemical analysis was performed in an ACQUITY UPLC I-Class System connected with a Xevo G2–XS QTOF mass spectrometer detector (Waters Co., Milford, MA, USA). Samples were separated on a Waters ACQUITY UPLC^®^ BEH C18 column (2.1 mm × 100 mm, 1.7 μm). The mobile phases were 2.5% acetic acid in water (A) and methanol (B), with a flowing elution gradient: 0–20 min, 5–25% B; 20–32 min, 25–55% B; 32–33 min, 55–90% B; 33–38 min, 90–55% B; 38–42 min, 55–5% B. The flow rate was 1 mL/min. UV spectra were set at 360 nm. The column oven and auto sampler temperatures were 35 °C and 10 °C, respectively.

For MS detection, the electrospray ionization conditions were set as follows: negative ion mode scanning in the range of 50–1000 Da; cone gas flow: 50 L/h; drying gas flow rate: 800 L/h, source temperature: 110 °C; drying gas temperature: 450 °C; nebulizer pressure: 45 psi; capillary voltage: 1 kV; cone voltage: 40 V.

### 4.4. Cell Culture, UVB Irradiation, and Sample Treatment

Normal human dermal fibroblast cells (NHDFs) were obtained by skin biopsy from a healthy young male donor (MCTT Bio, Inc., Seoul, Korea) [39]. The cells were cultured in DMEM containing 1% penicillin-streptomycin and 10% FBS at 37 °C in a 5% CO_2_ humidified incubator. When the cells reached 80–90% confluency, the NHDFs were subjected to UVB (144 mJ/cm^2^) irradiation using a Bio-Link BLX-312 (Vilber Lourmat GmbH, Vilber Lourmat, Marne-la-Vallée, France) [40]. Afterward, the cells were rinsed with phosphate-buffered saline (PBS) and immediately treated with E. officinalis (1 µg/mL, 10 µg/mL, or 50 µg/mL) in serum-free DMEM. Non-irradiated control cells were fed with serum-free DMEM medium.

### 4.5. Cell Viability and Light Microscopic Analysis

The viability of NHDFs treated with *E. officinalis* after UVB irradiation was evaluated using the MTT assay, as described previously [41]. NHDF cells (2 × 10^5^/35 mm^2^ dish) were exposed to UVB (144 mJ/cm^2^) and then treated with *E. officinalis* (or not for UVB-irradiated control cells). After 72 h of treatment, 1 mL of MTT (100 μg/mL) was added and incubated at 37 °C for 3 h. Then the MTT solution was discarded, and the purple formazan crystals were resolved in 800 µL of sterile DMSO. Finally, the dishes were shaken for 10 min at room temperature. The optical density values of 100-μL portions of formazan dissolved in DMSO were documented using a microplate reader (Molecular Devices FilterMax5; San Francisco, CA, USA).

### 4.6. Measurement of ABTS Radical Scavenging Activity

The radical scavenging capacity of *E. officinalis* extract was assessed using a previously published ABTS radical cation (ABTS•+) decolorization assay with small modifications to the method [40,42]. ABTS•+ was produced by reacting 7 mM of ABTS stock solution with 2.45 mM potassium persulfate (1:1) in phosphate buffered saline (PBS, 100 mM, pH 7.4); the solution was then stored in the dark at room temperature for 12–16 h before use. The ABTS•+ working solution was diluted with PBS to an absorbance of 0.65 ± 0.02 at 734 nm. The test was carried out using a Molecular Devices FilterMax5 microplate reader and a 96-well plate, with a total volume of 200 µL containing 196 µL of diluted ABTS+ solution and 4 µL of samples (final concentrations 1–250 µg/mL) in PBS. After 20 min of incubation, the absorbance of the reaction mixture was measured at 734 nm. Arbutin was used as a positive control.

### 4.7. Measurement of ROS Generation

To check the intracellular ROS levels, NHDF cells (2 × 10^5^/35 mm^2^ dish) were subjected to UVB (144 mJ/cm^2^) and provided with different concentrations of *E. officinalis*. After 24h, the NHDFs were collected and stained with an oxidant-sensing probe and 30 μM of 2’7’-dichloro-dihydro-fluorescein diacetate (DCFH-DA; Sigma-Aldrich, St. Louis, MO, USA). These cells were placed away from light for 30 min at room temperature and then inspected using flow cytometry with excitation at 490 nm and emission at 525 nm (FACSCaliburTM; Becton-Dickinson, San Jose, CA, USA).

### 4.8. Wound Healing Assay

To observe their wound-healing ability, NHDF cells (2 × 10^5^/well) were seeded in 6-well plates and grown to 80% confluence. Wounds were produced by scratching the monolayers with a 200-μL pipette tip and removing the cell debris with PBS. Afterward, cells were administered with different concentrations of *E. officinalis* and placed in a 37 °C incubator. The migration of cells at the edge of the wound was observed at 24 h, 48 h and 72 h. Then the cells were stained with 0.5% crystal violet (Sigma-Aldrich, St. Louis, MO, USA) and captured in a photograph at 72 h.

### 4.9. Hoechst 33258 Staining

Apoptosis in UVB-exposed NHDF cells following treatment with *E. officinalis* was evaluated by Hoechst 33258 staining. NHDFs (2 × 10^5^/well) were seeded onto sterile glass coverslips in 6-well plates. After UVB exposure, cells were administered with *E. officinalis* (1, 10, or 50 μg/mL) and incubated for 72 h. Cells were rinsed twice with PBS and then fixed in 4% paraformaldehyde for 10 min. Cells were then rinsed twice with PBS and dyed with 100 µL of Hoechst 33258 (10 μg/mL) solution and kept away from light for 10 min at room temperature. The morphologies of the apoptotic cells were viewed and captured by a fluorescence microscope (400×, Optinity, Korean Labtech, Seoul, Korea). The apoptotic rate was assessed as a percentage of apoptotic cells over the total number of cells in five randomly selected areas.

### 4.10. Determination of MMP-1, MMP-3, IL-6, TGF-β1, and Type I Procollagen

After 72 h of *E. officinalis* administration, commercial ELISA kits were used to measure the production of MMP-1, MMP-3, IL-6, TGF-β1, and type I procollagen in the culture supernatant. All of the experimental procedures were performed following manufacturer protocols.

### 4.11. Reverse Transcription-Polymerase Chain Reaction (RT-PCR)

Total RNA isolation was executed using a Trizol Reagent kit (Invitrogen Life Technologies, Carlsbad, CA, USA) and carefully following the manufacturer’s instructions [41]. First, 2 μg of RNA was used for cDNA synthesis with AccuPower HotStar RT PreMix, and the PCR reaction was completed using AccuPower HotStar PCR PreMix (Bioneer, Daejeon, Korea). The primers used in this study are listed in Table 1. The PCR products were divided by 2.0% agarose gel electrophoresis and detected by a UV transilluminator. Glyceraldehyde-3-phosphate dehydrogenase (GAPDH) was used as an internal control.

### 4.12. Western Blot Analysis

The influence of *E. officinalis* on the level of photoaging-related proteins was determined by Western blot analysis. After UVB exposure and *E. officinalis* treatment, NHDF cells were collected and lysed with RIPA lysis buffer. Protein concentrations were quantified using a Bradford assay kit (Sigma-Aldrich, St. Louis, MO, USA). Equal amounts (50 μg) of total protein were resolved by 10% sodium dodecyl sulfate polyacrylamide gel electrophoresis (SDS-PAGE) and applied to a nitrocellulose membrane. The membrane was shaken in 5% skim milk for 1 h and then incubated with primary antibody overnight at 4 °C. Subsequently, the membrane was incubated with horseradish peroxidase–linked secondary antibody for 1 h. Protein expression levels were detected using an ECL detection solution (Fujifilm, LAS-4000, Tokyo, Japan) and analyzed with ImageMasterTM 17 2D Elite software, version 3.1 (Amersham Pharmacia Biotech, Piscataway, NJ, USA).

### 4.13. Statistical Analysis

All of the data are presented as means ± standard deviation (SD). Statistical analyses used the one-way analysis of variance followed by Dunnett’s test. * *p* < 0.05, ** *p* < 0.01, and *** *p* < 0.001 were considered to be statistically significant.

## 5. Conclusions

In summary, the present study indicates that *E. officinalis* ethanol extract protected in vitro NHDF cells against UVB-induced photoaging by suppressing oxidative stress, cell apoptosis, inflammation, and the associated MAPK/AP-1 pathways. This preliminary study demonstrates the protective effects of *E. officinalis* against UVB-induced photoaging. However, the principal active components of the herb have not been quantified clearly. Further investigation of the phytochemical composition and content of *E. officinalis* and its function in photoaging in in vivo animal models is warranted.

## Figures and Tables

**Table 1:** List of primers used in this study.

Gene Name	Forward Primer (5′→3′)	Reverse Primer (5′→3′)
MMP-1	5′ATTCTACTGATATCGGGGCTTTGA3′	5′ATGTCCTTGGGGTATCCGTGTAG3′
Type I Procollagen	5′CTCGAGGTGGACACCACCCT3′	5′CAGCTGGATGGCCACATCGG3′
GAPDH	5′ACCACAGTCCATGCCATCAC3′	5′CCACCACCCTGTTGCTGTAG3′

## Supplementary Materials

Supplementary materials can be found at http://www.mdpi.com/1422-0067/19/11/3327/s1.

## References

[1] Naidoo K., Hanna R., Birch-Machin M.A. (2018). What is the role of mitochondrial dysfunction in skin photoaging?. Exp. Dermatol..

[2] Wittenauer J., Mäckle S., Sußmann D., Schweiggert-Weisz U., Carle R. (2015). Inhibitory effects of polyphenols from grape pomace extract on collagenase and elastase activity. Fitoterapia.

[3] Stenehjem J.S., Robsahm T.E., Bratveit M., Samuelsen S.O., Kirkeleit J., Grimsrud T.K. (2017). Ultraviolet radiation and skin cancer risk in offshore workers. Occup. Med..

[4] Ichihashi M., Ando H., Yoshida M., Niki Y., Matsui M. (2009). Photoaging of the skin. Anti-Aging Med..

[5] Silveira J.E.P.S., Pedroso D.M.M. (2014). UV light and skin aging. Rev. Environ. Health.

[6] Ichihashi M., Ando H. (2014). The maximal cumulative solar UVB dose allowed to maintain healthy and young skin and prevent premature photoaging. Exp. Dermatol..

[7] Gaiba S., Tucci-Viegas V.M., Franca L.P., Lasakosvitsch F., Azevedo F.L., Moraes A.A., Ferreira A.T., Franca J.P. (2012). Biological effects induced by ultraviolet radiation in human fibroblasts. Flow Cytometry-Recent Perspectives.

[8] Son Y., Kim S., Chung H.T., Pae H.O. (2013). Reactive oxygen species in the activation of MAP kinases. Methods Enzymol..

[9] Barr R.K., Bogoyevitch M.A. (2001). The c-Jun N-terminal protein kinase family of mitogen-activated protein kinases (JNK MAPKs). Int. J. Biochem. Cell Biol..

[10] Klein T., Bischoff R. (2011). Physiology and pathophysiology of matrix metalloproteases. Amino Acids.

[11] Xu H., Yan Y., Li L., Peng S., Qu T., Wang B. (2010). Ultraviolet B-induced apoptosis of human skin fibroblasts involves activation of caspase-8 and -3 with increased expression of vimentin. Photodermatol. Photoimmunol. Photomed..

[12] Lee C.-H., Wu S.-B., Hong C.-H., Yu H.-S., Wei Y.-H. (2013). Molecular mechanisms of UV-induced apoptosis and its effects on skin residential cells: The implication in UV-based phototherapy. Int. J. Mol. Sci..

[13] Bielory L., Heimall J. (2003). Review of complementary and alternative medicine in treatment of ocular allergies. Curr. Opin. Allergy Clin. Immunol..

[14] Fraunfelder F.W. (2004). Ocular side effects from herbal medicines and nutritional supplements. Am. J. Ophthalmol..

[15] Petrichenko V., Sukhinina T., Babiyan L., Shramm N. (2006). Chemical composition and antioxidant properties of biologically active compounds from Euphrasia brevipila. Pharm. Chem. J..

[16] Singh H., Du J., Singh P., Yi T.H. (2018). Ecofriendly synthesis of silver and gold nanoparticles by *Euphrasia officinalis* leaf extract and its biomedical applications. Artif. Cells Nanomed. Biotechnol..

[17] Porchezhian E., Ansari S.H., Shreedharan N.K. (2000). Antihyperglycemic activity of Euphrasia officinale leaves. Fitoterapia.

[18] Dimitrova M., Hristova L., Damianova E., Yordanova Y., Petrova N., Kapchina-Toteva V. (2014). Antioxidant activity and secondary metabolites in different extracts of *Euphrasia officinalis* L. growing in Bulgaria. Sci. Technol. Med..

[19] Paduch R., Woźniak A., Niedziela P., Rejdak R. (2014). Assessment of eyebright (*Euphrasia officinalis* L.) extract activity in relation to human corneal cells using in vitro tests. Balkan Med. J..

[20] Novy P., Davidova H., Serrano-Rojero C.S., Rondevaldova J., Pulkrabek J., Kokoska L. (2015). Composition and Antimicrobial Activity of Euphrasia rostkoviana Hayne Essential Oil. Evid. Based Complement. Altern. Med..

[21] Bigagli E., Cinci L., D’Ambrosio M., Luceri C. (2017). Pharmacological activities of an eye drop containing *Matricaria chamomilla* and *Euphrasia officinalis* extracts in UVB-induced oxidative stress and inflammation of human corneal cells. J. Photochem. Photobiol. B.

[22] Blazics B., Alberti Á., Kursinszki L., Kéry Á., Béni S., Tölgyesi L. (2011). Identification and LC-MS-MS determination of acteoside, the main antioxidant compound of Euphrasia rostkoviana, using the isolated target analyte as external standard. J. Chromatogr. Sci..

[23] Rajendram R., Preedy V.R., Patel V.B. (2015). Branched Chain Amino Acids in Clinical Nutrition.

[24] Philips N., Siomyk H., Bynum D., Gonzalez S. (2014). Skin cancer, polyphenols, and oxidative stress. Cancer.

[25] Sun Z., Park S.Y., Hwang E., Zhang M., Seo S.A., Lin P., Yi T.H. (2017). Thymus vulgaris alleviates UVB irradiation induced skin damage via inhibition of MAPK/AP-1 and activation of Nrf2-ARE antioxidant system. J. Cell. Mol. Med..

[26] Rafaela T., Silva L.R. (2013). Bioactive compounds and in vitro biological activity of *Euphrasia rostkoviana* Hayne extracts. Ind. Crops Prod..

[27] Daniela G., Vincenzo D.F. (2017). Euphrasia rostkoviana Hayne-Active Components and Biological Activity for the Treatment of Eye Disorders.

[28] Ellery D. (2017). Herbal Formulation for Treating Chronic Fatigue Syndrome.

[29] Sun Z., Park S.Y., Hwang E., Park B., Seo S.A., Cho J.-G., Zhang M., Yi T.-H. (2016). Dietary Foeniculum vulgare Mill extract attenuated UVB irradiation-induced skin photoaging by activating of Nrf2 and inhibiting MAPK pathways. Phytomedicine.

[30] Salucci S., Burattini S., Battistelli M., Baldassarri V., Maltarello M.C., Falcieri E. (2012). Ultraviolet B (UVB) irradiation-induced apoptosis in various cell lineages in vitro. Int. J. Mol. Sci..

[31] Subedi L., Lee T.H., Wahedi H.M., Baek S.-H., Kim S.Y. (2017). Resveratrol-enriched rice attenuates UVB-ROS-induced skin aging via downregulation of inflammatory cascades. Oxid. Med. Cell. Longev..

[32] Li W., Fan J., Chen M., Guan S., Sawcer D., Bokoch G.M., Woodley D.T. (2004). Mechanism of human dermal fibroblast migration driven by type I collagen and platelet-derived growth factor-BB. Mol. Biol. Cell.

[33] Kulka M. (2013). Mechanisms and treatment of photoaging and photodamage. Using Old Solutions to New Problems-Natural Drug Discovery in the 21st Century.

[34] Campo G.M., Avenoso A., Campo S., D’Ascola A., Ferlazzo A.M., Sama D., Calatroni A. (2006). Purified human chondroitin-4-sulfate reduced MMP/TIMP imbalance induced by iron plus ascorbate in human fibroblast cultures. Cell Biol. Int..

[35] Van Doren S.R. (2015). Matrix metalloproteinase interactions with collagen and elastin. Matrix Biol..

[36] Hwang E., Kim S.H., Lee S., Lee C.H., Do S.G., Kim J., Kim S.Y. (2013). A comparative study of baby immature and adult shoots of Aloe vera on UVB-induced skin photoaging in vitro. Phytother. Res..

[37] Kim J.M., Noh E.M., Song H.K., Lee G.S., Kwon K.B., Lee Y.R. (2018). Reversine inhibits MMP-1 and 3 expressions by suppressing of ROS/MAPK/AP-1 activation in UV-stimulated human keratinocytes and dermal fibroblasts. Exp. Dermatol..

[38] Sun Z., Park S.Y., Hwang E., Zhang M., Jin F., Zhang B., Yi T.H. (2015). Salvianolic Acid B Protects Normal Human Dermal Fibroblasts Against Ultraviolet B Irradiation-Induced Photoaging Through Mitogen-Activated Protein Kinase and Activator Protein-1 Pathways. Photochem. Photobiol..

[39] Lee E., Kim D.Y., Chung E., Lee E.A., Park K.-S., Son Y. (2014). Transplantation of cyclic stretched fibroblasts accelerates the wound-healing process in streptozotocin-induced diabetic mice. Cell Transplant..

[40] Hwang E., Ngo H.T., Seo S.A., Park B., Zhang M., Yi T.-H. (2018). Protective effect of dietary Alchemilla mollis on UVB-irradiated premature skin aging through regulation of transcription factor NFATc1 and Nrf2/ARE pathways. Phytomedicine.

[41] Hwang E., Park S.Y., Lee H.J., Lee T.Y., Sun Z.W., Yi T.H. (2014). Gallic acid regulates skin photoaging in UVB-exposed fibroblast and hairless mice. Phytother. Res..

[42] Li X., Wang Z., Wang L., Walid E., Zhang H. (2012). In vitro antioxidant and anti-proliferation activities of polysaccharides from various extracts of different mushrooms. Int. J. Mol. Sci..

