# Supplementary material for: Protective Effects of Euphrasia officinalis Extract against Ultraviolet B-Induced Photoaging in Normal Human Dermal Fibroblasts
Source: Int J Mol Sci. 2018 Oct 25;19(11):3327. doi: 10.3390/ijms19113327 (PMC6275060; doi:10.3390/ijms19113327)
Supplement: Supplementary file 1 [file ijms-19-03327-s001.pdf]

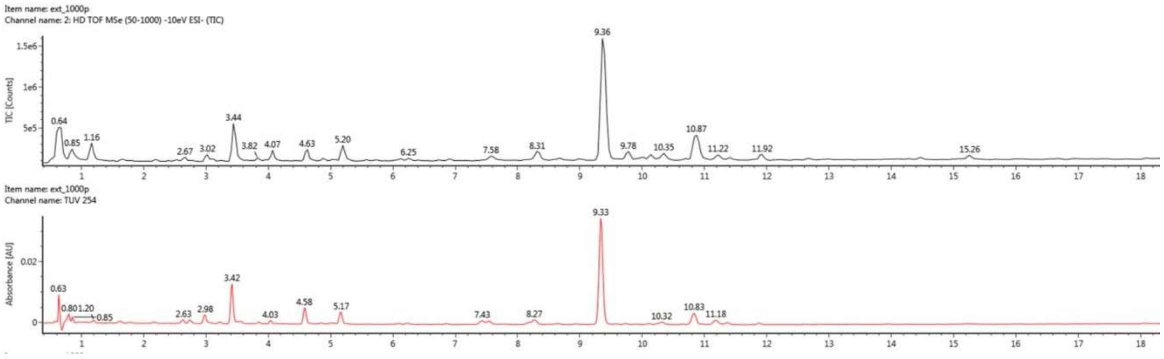

Figure S1. The identified components in *E. officinalis* ethanol extract using UPLC-QTOF-MS.

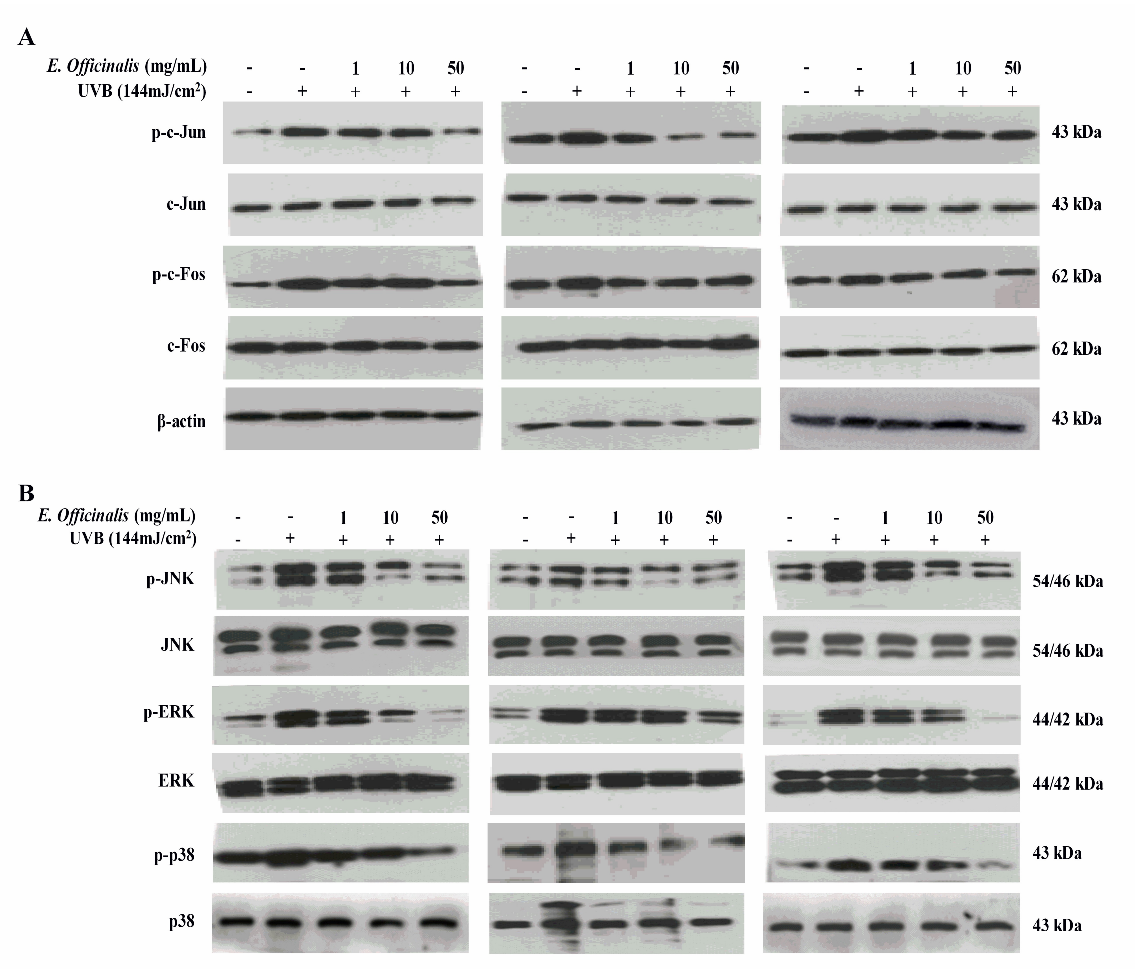

Figure S2. The original Western blot images related to this article in triplicate. (**A**) Effect of E. officinalis on activator protein 1 (AP-1) signaling–related proteins; (**B**) Effect of E. officinalis on mitogen-activated protein kinase (MAPK) signaling–related proteins.

Table S1. QTOF-MS data of identified components in *E. officinalis* ethanol extract.

Formula	Neutral Mass (Da)	Observed Neutral Mass (Da)	Observed *m*/*z*	Mass Error (mDa)	Mass Error (ppm)	Observed RT (min)	Observed CCS (Å²)	Detector Counts	Response	Adducts	Identification
$C_9H_8O_4$	180.0423	180.042	179.035	−0.2	−1.3	2.78	218.12	1117	1024	-H	Caffeic acid
$C_{27}H_{30}O_{16}$	610.1534	610.1538	609.147	0.4	0.6	7.59	231.96	3345	2548	-H	Rutin
$C_{21}H_{20}O_{11}$	448.1006	448.1007	447.093	0.1	0.2	8.3	210.51	7564	6015	-H	Luteolin-glucoside
$C_{29}H_{36}O_{15}$	624.2054	624.2045	623.197	−0.9	−1.4	9.38	265.76	17,677	13,942	-H	Acteoside
